# Iranian midwives’ awareness and performance of respectful maternity care during labor and childbirth

**DOI:** 10.18332/ejm/143873

**Published:** 2021-12-27

**Authors:** Simin Haghdoost, Fatemeh Abdi, Azam Amirian

**Affiliations:** 1 Department of Midwifery and Reproductive Health, Shahid Beheshti University of Medical Sciences, Tehran, Iran; 2 School of Nursing and Midwifery, Alborz University of Medical Sciences, Karaj, Iran; 3 Department of Midwifery, School of Nursing and Midwifery, Jiroft University of Medical Sciences, Jiroft, Iran

**Keywords:** performance, awareness, respectful maternity care, midwives, childbirth

## Abstract

**INTRODUCTION:**

Midwives’ perceptions of Respectful Maternity Care (RMC) play an important role in promoting quality of care. This study aimed to explore the awareness and performance of Iranian midwives of RMC during childbirth.

**METHODS:**

A cross-sectional study was carried out from November to December 2020 to evaluate 130 midwives’ awareness and performance of RMC during childbirth at four public hospitals in Urmia province, Iran. Participants were midwives who were working in the labor unit and had at least one year of work experience. The Midwives’ Knowledge and Practice Scale on Respectful Maternity Care was used to assess midwives’ awareness and performance. The quality assessment of questionnaires was based on the mean for each item. A multivariate linear regression approach was developed to evaluate the relationship between midwives’ age, academic education level plus occupational information and their awareness and performance of RMC.

**RESULTS:**

This study revealed that Iranian midwives had good awareness but fair performance of RMC. The mean scores of the overall awareness and performance of RMC were 36.07±10.13 and 75.47±35.4, respectively. Midwives’ performance on two domains was fair including ‘Giving emotional support’ and ‘Providing safe care’. The results of multivariate linear regression analysis showed a significant negative relationship between job satisfaction and midwives’ performance on RMC. Also work experience plus a Master’s degree in midwifery had positive significant effects on midwives’ awareness along with performance on RMC (p<0.05).

**CONCLUSIONS:**

Promoting respectful maternity care requires essential interpersonal and communication skills and supportive attitudes from midwives.

## INTRODUCTION

Pregnancy and childbirth are momentous events in the lives of women and families. Women’s experiences with caregivers at this time have the impact to empower and comfort or to inflict lasting damage and emotional trauma^1^. Disrespect and abuse of women in maternal healthcare is a prevalent and serious issue in many countries^1^. A review by Bohren et al.^2^ found evidence of all seven types of disrespect and abuse of women during childbirth across all geographical regions and income-level settings, but were manifested in different forms depending on the context. Moreover, maltreatment during childbirth reduces women’s satisfaction, which is an important factor to reduce women’s confidence to providers in maternity services^3^.

Respectful maternity care (RMC) is a universal human right of childbearing women (RMC Charter) and refers to seven domains that have been developed by White Ribbon Alliance (WRA)^4^. The RMC charter has been used as a tool to educate health workers about maternity care and human rights^1^.

Women who receive respectful maternity care may be more likely to give birth vaginally and be satisfied and have a shorter labor^5^. This is because continuous midwives’ support for women during childbirth is an important part of respectful maternity care^6^. This support includes emotional support (continuous presence, touching, empathy, reassurance, and praise) and information about labor progress. It may also include advice about coping techniques as well as comfort measures^7^.

Iran is one of the 9 countries which achieved the fifth Millennium Development Goal (MDG) target of reducing the maternal mortality ratio by at least 75% from 1990 to 2015, mostly due to increased birth facilities and skilled birth attendants^8,9^. However, challenges are facing the country in relation to promoting maternal care quality. Improving the quality of childbirth care is an essential component of strategies to reach the third Sustainable Development Goal (SDG3) by 2030^10^. To address this issue, the Ministry of Health and Medical Education (MOHME) of Iran has also made great efforts to develop the Bill of Rights of pregnant women^11^. Midwives’ awareness of principles of RMC has been increased but it is not comprehensive^12^. A review of existing studies conducted on women’s experience of childbirth in Iran showed that women experience some forms of disrespect and carelessness, persistent negative memory of pain and persistent fear of childbirth^13-15^. Furthermore, all these reported issues, in turn, were associated with the increased demand for elective caesarean birth in Iran. Hence, 50–60% of births are caesarean sections (CS) in Iran^16^. Evidence suggests that most women would improve their utilization of vaginal delivery in the maternity care system if midwives showed respectful behavior^13,17^.

The first step to promote RMC is to evaluate the awareness and performance of the RMC providers. With this aim, this study evaluated midwives’ awareness and performance of RMC principles during labor and childbirth in northwest of Iran.

## METHODS

### Design and setting

This cross-sectional study was conducted to evaluate midwives’ awareness and performance of RMC during labor and birth from November to December 2020 at four public hospitals in Urmia province, Iran.

### Sample and recruitment

The study population (130) consisted of all midwives working in four public hospitals in a northwestern province of Iran. All midwives working in the study hospitals were invited to participate in the survey. The participants who agreed and signed the informed consent, participated in the study. Regarding the inclusion criteria, the participants in this study were midwives who were working in the labor unit, had a midwifery academic degree of education, and at least one year’s work experience. The participants’ midwives who had less than one year’s work experience were excluded (9 midwives). Midwives were selected by the census sampling method and finally, among 139 midwives working in four public hospitals, 130 midwives were included and 9 midwives were excluded from the study. The interviews were conducted in convenient places in public hospitals. Data collection tools included a questionnaire to record demographics, and a Midwives’ Knowledge and Practice Scale on Respectful Maternity Care (MKP-RMC)^18^ to assess midwives’ awareness and performance were handed to them.

### Demographic questionnaire

The demographic questionnaire consisted of two sections: 1) demographic data such as age, marital status, academic degree in midwifery, and 2) occupational information including work experience, job satisfaction, interest in the field of study, workplace, working shifts, and employment status.

### Midwives’ knowledge and practice scale on respectful maternity care (MKP-RMC)

We used the Midwives’ Knowledge and Practice Scale on Respectful Maternity Care (MKP-RMC) as a valid and reliable tool for measuring midwives’ knowledge and practice of respectful care during labor and childbirth. The scale has two sections (Knowledge and Practice) and has been designed based on three domains^18^. This scale has 23 items in knowledge and 23 items in practice section that covered the three domains: giving emotional support, providing safe care, and preventing mistreatment. The items’ numbers in domains of awareness sections were 12 (giving emotional support), 8 (providing safe care), and 3 (preventing mistreatment). The items’ numbers in domains of performance sections were 11 (giving emotional support), 9 (providing safe care), and 3 (preventing mistreatment). The awareness scale is categorized as: ‘correct’, ‘I don’t know’, and ‘incorrect’, and scored as 2, 1, and 0, respectively. The performance scale consists of items on a 5-point Likert scale, and categorized as: 5=‘always’, 4=‘often’, 3=‘sometimes’, 2=‘rarely’, and 1=‘never’. The means of awareness and performance are calculated based on the number of items in each domain. Finally, the scores above the mean are dichotomized as ‘good’ and ‘fair’, and the scores less than the mean are considered ‘weak’. A composite score is then created by summing up all the individual items within each scale. The highest scores of awareness and performance are 24 and 115, and the lowest scores are 0 and 23, respectively. The validity of the Persian version of this questionnaire has been measured by Moridi et al.^18^. To determine the reliability of the instrument, internal consistency and test-retest methods were used. In order to calculate the Cronbach’s alpha coefficient, the questionnaire was completed by 15 midwives. The obtained scores of the Cronbach’s alpha coefficient of awareness and performance items were 0.78 and 0.87, respectively, indicating a desired correlation between the items. In addition, 15 midwives completed the questionnaire twice with a 2-week interval in order to examine the stability of the scale by calculating the Intra-class Correlation Coefficient (ICC). An ICC of ≥0.8 was considered acceptable.

### Data analysis

The data were analyzed using IBM SPSS software (version 24)^19^. Descriptive statistics, central and scatter indices such as percentage, mean and standard deviation, were used. A multivariate linear regression model was developed to investigate the relationship among midwives’ awareness and performance with age, work experience, academic degree in midwifery, job satisfaction, work place, job selection by interest, employment status and working shifts. The significance level was set at p<0.05.

## RESULTS

### Demographics

In all, 139 midwives were selected by census sampling method in four public hospitals in a northwestern province of Iran. Finally, 130 midwives were included in the study. Table 1 presents the demographic characteristics of participant midwives. The mean age was 32.2 years and 50.0% were aged 31–40 years. Majority of midwives were married, had a Bachelor’s degree in midwifery (88.7%) and worked in the labor unit (73.9%) in rotational shifts (70.3%). Their average work experience was 5.42 years. The majority (89.5%) had entered their job selection by interest and a third (35.9%) were completely satisfied with their jobs.

**Table 1:** Characteristics of participant midwives working in four public hospitals in Urmia, Iran, 2020 (N=130)

*Characteristics*	*n (%)*
**Age** (years)	
21–30	49 (37.7)
31–40	65 (50.0)
41–50	16 (12.3)
**Academic degree in midwifery**	
Associate’s	4 (3.7)
Bachelor’s	113 (88.7)
Master’s	13 (7.6)
**Marital status**	
Married	70 (52.3)
Single	60 (47.7)
**Work experience** (years)	
1–7	84 (64.8)
8–15	34 (25.8)
16–23	8 (6.3)
≥24	3 (3.1)
**Employment status**	
Official	32 (24.2)
Contractual	69 (50.8)
Compulsory service program	29 (25)
**Work place**	
Labor unit	97 (73.9)
Admission unit	33 (26.1)
**Working shifts**	
Morning	15 (11.7)
Evening	13 (10.2)
Night	10 (7.5)
Rotation	92 (70.3)
**Job satisfaction**	
Completely satisfied	47 (35.9)
Somewhat satisfied	46 (35.2)
Dissatisfied	37 (28.9)
**Job selection by interest**	
Yes	116 (89.5)
No	14 (10.5)

According to Table 2, the mean scores of the overall Iranian midwives’ awareness and performance on RMC were 36.07±10.13 and 75.47±35.4, respectively. The quality assessment based on the mean for overall awareness and performance on RMC showed that Iranian midwives had good awareness and fair performance. The participating midwives had scored good on both performance and awareness in the domain of ‘Preventing mistreatment’. They had also good awareness and fair performance in the ‘Giving emotional support’ and ‘Providing safe care’ domains, respectively.

**Table 2:** Survey of midwives’ awareness and performance of RMC in related domains working in four public hospitals in Urmia, Iran, 2020 (N=130)

*Domains*	*Section*	*Mean ± SD*	*n (%)*		
			*Good*	*Fair*	*Weak*
**Overall**	Awareness	36.07±10.13	64 (49.2)	52 (40)	14 (10.8)
	Performance	75.47±31.4	40 (30.8)	67 (51.5)	23 (17.7)
**Giving emotional support**	Awareness	18.5±5.68	62 (47.7)	58 (44.6)	10 (7.7)
	Performance	30.63±15.97	25 (19.2)	86 (66.2)	19 (14.6)
**Providing safe care**	Awareness	12.2±3.82	65 (50)	56 (43.1)	9 (6.9)
	Performance	29.34±13.66	41 (31.5)	77 (52.2)	12 (9.2)
**Preventing mistreatment**	Awareness	5.37±0.63	68 (52.3)	57 (43.8)	5 (3.9)
	Performance	11.25±1.77	68 (52.3)	55 (42.3)	7 (5.4)

According to Table 3, the highest and lowest mean scores in the first domain (giving emotional support) of performance section were in the items ‘I welcome laboring woman warmly’ and ‘I establish friendly and appropriate relationship with the laboring woman’, respectively. The highest and lowest mean scores in the second domain (providing safe care) of performance section were in the items ‘I keep medical records and the results of examinations and consultations confidential’ and ‘I provide clear information about progress of labor’, respectively. The highest and lowest mean scores in the third domain (preventing mistreatment) of performance section were in the items ‘I may not shout at laboring woman if she does not cooperate’ and ‘I do allow the laboring woman to have a companion inside the labor unit’, respectively (Table 3).

**Table 3:** Midwives’ performance of RMC during childbirth in Urmia, Iran, 2020 (N=130)

*Domain/items*	*Midwives’ performance Mean ± SD*
**Domain 1: Giving emotional support**	
1. I welcome laboring woman warmly.	4.51±0.49
2. I introduce myself to the laboring woman.	3.41±0.59
3. I show the laboring woman around the labor unit.	3.16±2.84
4. I establish friendly and appropriate relationship with the laboring woman.	2.34±1.66
5. I support laboring woman by encouraging and calming touch.	3.06±1.94
6. I use the name preferred by a laboring woman.	3.36±0.64
7. I am continuously or timely available beside her.	3.03±2.97
8. I provide laboring woman with correct and clear information about the care, interventions and progress of labor.	3.32±0.63
9. I build friendly relationship in a way that she feels comfortable to ask her questions.	3.23±1.77
10. I provide a comfortable environment for laboring woman.	3.33±1.67
11. I support laboring woman to be in her desired birthing position.	3.2±1.8
**Domain 2: Providing safe care**	
1. I keep medical records and the results of examinations and consultations confidential.	4.33±0.67
2. I cover the laboring woman’s body during examinations, using sheets.	2.23±1.87
3. I perform all interventions with laboring woman’s informed consent.	2.03±1.97
4. I provide equal care to all women, regardless of their socio-economic status, ethnicity, etc.	4.27±0.73
5. I support laboring woman to take care of herself and her baby.	4.11±0.89
6. I provide evidence-based and up-to-date childbirth care.	2.86±2.14
7. I pay attention to laboring woman’s safety in providing care and interventions.	4.27±0.73
8. I respect beliefs and culture of laboring woman and her companion.	3.33±1.67
9. I provide clear information about progress of labor.	2.01±1.99
**Domain 3: Preventing mistreatment**	
1. I do allow the laboring woman to have a companion inside the labor unit.	1.89±1.13
2. I do not beat the laboring woman if she does not cooperate.	4.59±0.41
3. I do not shout at the laboring woman if she does not cooperate.	4.77±0.23

According to Table 4, the highest and lowest mean scores in the first domain (giving emotional support) of awareness section were in the items ‘Calling laboring woman’s name as she desires’ and ‘Freedom in choosing birthing position’, respectively. The highest and lowest mean scores in the second domain (providing safe care) of awareness section were in the items ‘Keeping medical records and the results of tests and consultations confidential’ and ‘Obtaining informed consent before performing any care and interventions’, respectively.

**Table 4:** Survey of midwives’ awareness of RMC during Childbirth working in four public hospitals in Urmia, Iran, 2020 (N=130)

*Domain/items*	*Midwives’ awareness Mean ± SD*
**Domain 1: Giving emotional support**	
1. Warm welcoming in entering to labor unit.	1.43±0.6
2. Showing around maternity labor unit’s environment.	1.8±0.3
3. Establishing friendly communication.	1.47±0.53
4. Encouraging and giving calming touch.	1.6±0.4
5. Calling laboring woman’s name as she desires.	1.76±0.24
6. Providing accurate and clear information about progress of labor, received care and interventions.	1.56±0.44
7. Providing friendly environment to ask questions.	1.55±0.43
8. Providing comfortable and calming environment.	1.71±0.29
9. Freedom in choosing birthing position.	1.24±0.76
10. Having companion of choice upon request.	1.35±0.65
11. Respecting laboring woman’s and her companion’s beliefs and culture.	1.56±0.44
12. Providing appropriate environment for companions.	1.4±0.6
**Domain 2: Providing safe care**	
1. Continuous or timely presence beside.	1.56±0.44
2. Keeping medical records and the results of tests and consultations confidential.	1.85±0.15
3. Obtaining informed consent before performing any care and interventions.	1.31±0.69
4. Providing equal care to all laboring woman regardless of their socio-economic status, ethnicity, etc.	1.45±0.55
5. Providing evidence-based and up-to-date childbirth care.	1.35±0.67
6. Providing pain relief.	1.59±0.41
7. Paying attention to safety in providing care and interventions.	1.68±0.32
8. Providing accurate information about progress of labor to companions.	1.42±0.58
**Domain 3: Preventing mistreatment**	
1. Attendance of unnecessary person during performing procedure.	1.74±0.26
2. Physical violence in the case of non-cooperation.	1.83±0.17
3. Shouting at the laboring woman in case of non-cooperation.	1.8±0.2

The highest and lowest mean scores in the third domain (preventing mistreatment) of awareness section were in the items including ‘Physical violence in the case of non-cooperation’ and ‘Attendance of unnecessary person during performing procedure’, respectively (Table 4).

A multivariate linear regression model was used to assess of the relationship between midwives’ awareness as well as performance with the important factors: age, work experience, academic degree in midwifery, job satisfaction, work place, job selection by interest, employment status, and working shifts. The results showed that work experience plus a Master’s degree in midwifery had positive significant effects on midwives’ awareness along with performance. Also, there was a significant negative relationship between job satisfaction and midwives’ performance. In this regard, increase in a year of work experience, was associated with a 0.968-point rise in midwives’ awareness on RMC. Midwives with a Master’s degree in the midwifery education had a 2.41-point rise in awareness on RMC compared to the midwives with an Associate’s degree in midwifery education. Additionally, increase in a year of work experience was associated with a 1.18-point rise in midwives’ performance on RMC. Midwives with a Master’s degree in the midwifery education had a 0.59-point rise in performance on RMC compared to the midwives with an Associate’s degree in midwifery education. However, a degradation in job satisfaction was associated with a 1.27-point decrease in midwives’ performance on RMC (Table 5).

**Table 5:** Multiple linear regression analyses between the combination of the factors with Iranian midwives’ awareness and performance on RMC

*Regression factors*	*Not standardized coefficients*		*Standardized coefficient B*	*t*	*p*
	*Coefficient B regression*	*Standard error*			
***Awareness***					
**Work experience**	0.968	0.274	1.617	0.320	0.03
**Age**	-0.280	0.338	-0.813	-0.137	0.210
**Academic degree in the midwifery**					
Master’s	2.41	2.18	2.11	0.167	0.036
Bachelor’s	1.37	1.01	0.1	1.33	0.098
Associate’s	0.173	1.26	0.01	0.137	0.89
**Job satisfaction**	-0.891	2.24	0.99	-0.037	0.41
**Employment status**	0.54	1.02	0.508	0.04	0.612
**Job selection by interest**	0.109	0.043	1.607	0.240	0.09
**Working shifts**	-0.063	0.042	-1.47	-0.137	0.142
**Work place**	-0.066	0.075	0.886	-0.065	0.37
**Employment status**	-0.063	0.07	0.901	-0.065	0.369
***Performance***					
**Work experience**	1.18	0.260	3.279	0.433	0.002
**Age**	0.1	0.330	0.255	0.032	0.7
**Academic degree in the midwifery**					
Master’s	0.59	0.26	2.21	0.175	0.028
Bachelor’s	0.3	0.51	0.1	1.6	0.109
Associate’s	0.23	0.188	0.09	1.26	0.207
**Job satisfaction**	-1.27	0.53	-2.41	-0.19	0.017
**Employment status**	-0.15	0.54	0.283	0.21	0.77
**Job selection by interest**	0.167	0.068	1.454	0.180	0.051
**Working shifts**	-0.076	0.074	-1.024	-0.075	0.307
**Work place**	-0.066	-0.71	0.934	-0.069	0.351
**Employment status**	-0.085	0.083	-1.027	-0.076	0.307

## DISCUSSION

This study revealed that Iranian midwives had good awareness of RMC but their awareness was not comprehensive on the number of items in each domain and their performance of RMC was fair. The first domain of our questionnaire, ‘giving emotional support’, showed that the participating midwives had a good awareness and fair performance. One of the items of this domain was ‘I establish friendly and appropriate relationship with the laboring woman’ which had the lowest mean score in the performance sections. Previous studies in Iran showed the need to improve midwives’ communication skills for providing better relationships with women during childbirth^15,20^. Lack of emotional support skills from midwives (included touching, empathy, compassion, reassurance taking mother’s hands, maintaining eye contact, creating a sense of trust and confidence, reduction of fear during labor) is one of the reasons that Iranian women have negative feelings due to pain, fear, and suffering, which would hinder their psychological development in labor wards^21,22^. The importance of this domain and its items are supported by other studies showing the key role of emotional supportive care by midwives. This would help mothers to relax and control their emotions and fears, and decrease the intensity of pain, shorten the duration of labor, and reduce caesarean birth, instrumental vaginal birth, use of any analgesia, lower the five-minute Apgar score as well as negative feelings about childbirth experiences^7,23^. Thus, care providers’ training in interpersonal care and emotional support could improve providers’ awareness and sensitize them on how to better offer counseling for women during childbirth, which will improve women’s birthing experience.

The second domain in the questionnaire was ‘providing safe care’. This domain indicates the participant midwives have had a good awareness and fairly good performance. One of the items of this domain is ‘providing informed consent before performing any care and interventions’ which had the lowest mean score in both awareness and performance sections. According to a review of the existing literature in Iran, Iranian women reported that the staff did not always ask permission to carry out labor and childbirth procedures. They experienced non-consented care such as labor augmentation, frequent vaginal examinations, and episiotomy as well as fundal pressure during childbirth^13,14,24^. This is because of medical dominance in the maternity care system and the lack of midwifery-led care in managing vaginal childbirth in Iran, whereby women cannot freely participate in the decision-making about their care^14^. Iranian midwives are trained and licensed according to international standards. However, their role is limited to managing vaginal childbirth in public hospitals^14^. In Iranian public hospitals, obstetricians responsible for all births and obstetric residents manage all vaginal deliveries, and midwives are less involved in vaginal childbirth^14^. Iran’s health system has an important effect on medicalization and thus needs to play a major role in changing the practice.

In our study, the participating midwives had a good awareness but a weak performance to ‘provide clear information about progress of labor’ in the second domain. A recent qualitative study from Iran on midwives’ perspectives of RMC also reported that most midwives considered involving women in the care process and decision making as an essential part of the RMC^12^. However, previous studies from Iran show that women did not receive adequate information about the process of labor, procedures, coping techniques and intervention procedures during childbirth that would prepare them to make a decision about their care^14,15^. This domain indicates that providing information regarding procedures (advice about coping techniques, comfort measures and medical intervention, etc.) and supporting her decision-making are essential components for improving the quality of care and birth satisfaction in Iran^25^.

In this study, midwives had a good awareness but a weak performance to ‘provide evidence-based and up-to-date childbirth care’ of the second domain. Iranian midwives are informed about evidence-based practice in childbirth as the Ministry of Health and Medical Education (MOHME) has included evidence-based care into the guidelines for management of vaginal birth in Iran^26^. However, Iranian women experience a high rate of unnecessary interventions such as early admission in labor, stimulation, induction of labor, episiotomy, frequently vaginal examinations, and caesarean section^13,14^. This is because within in the Iranian birth context, midwives are not autonomous when they look after women in labor and they are not involved in any decision-making process; the care they provide is under the supervision of the obstetrician in charge^14^. Besides, regarding medical dominance in the Iranian maternity care system, obstetricians tend to have some unnecessary interventions during vaginal childbirth in low-risk women, which may increase the rate of cesarean birth and ectopic scars^14^. However, there is strong evidence that the decrease of interventions during vaginal birth in low-risk women can make childbirth safer and improve the quality of obstetric care^27^. In order to provide good quality maternity care, health workers require a well-functioning health system structure which supports evidence-based practice.

The third domain in the questionnaire is ‘Preventing mistreatment’. This domain indicates the participating midwives had a good mean score on both preferences and awareness. In accordance with these results, a number of qualitative studies in Iran show that pregnant women reported that midwives fulfilled all their needs in respectful manner; they even provided equal services for the entire term^28-30^. One of the items of this domain is ‘I do not allow the laboring woman to have companion inside the labor unit’ had the lowest mean score in performance sections. This is because, presence of women with their companion is prohibited in public hospitals in Iran^15^. A review of the existing literature in Iran also shows midwives who do not necessarily consider mothers’ emotional needs, laboring alone was extremely difficult for many women and reported that negative feelings during labor^15,23^. Although some certain religious issues have legally prohibited husbands from attending delivery room in Iran, friends and female relatives are good choices for mothers to trust these companions and feel comfortable around them^31^. Based on World Health Organization's (WHO) recommendations, healthcare administrators should facilitate the presence of a companion during labor^32^. Our findings also suggest that the health system provides the structure where mothers can access good quality care.

The results of multivariate linear regression analysis showed that there was a significant negative relationship between job satisfaction and midwives’ performance on RMC. Work experience plus a Master’s degree in midwifery had positive significant effects on midwives’ awareness along with performance on RMC. In accordance with these results, previous studies from Iran also showed that more experienced midwives have good relationships with women during childbirth and have a positive attitude towards the rights of pregnant women^30,33^. A study conducted in Iran showed that the midwives with a Master’s degrees displayed greater knowledge or skills and practice of evidence-based practice, which have a more positive attitude towards the rights of pregnant women than those with a Bachelor’s degree^34^. A review of studies from Iran and Korea indicated that there was a good relationship between job satisfaction and job performance of midwives^35,36^. Midwives, as main caregivers, would have undertaken the vaginal delivery for women at low risk. It would have a positive impact on the job satisfaction of midwifery. Therefore, it is recommended that health system provide organizational support for Iranian midwives

### Limitations

The evaluation of midwives’ performance was based on midwives’ self-administered tool. To overcome this limitation, an observational study is recommended.

## CONCLUSIONS

This study has demonstrated that Iranian midwives knew the principles of RMC but their awareness was not comprehensive on the number of items in each domain and their performance of RMC was fair. Midwives’ performance on giving emotional support and providing safe care were fair. The results of linear regression analysis showed that there was a significant negative relationship between job satisfaction and midwives’ performance on RMC. Also, work experience plus a Master’s degree in midwifery had positive significant effects on midwives' awareness along with performance on RMC. The main goal should be to include interventions such as providing midwife-led models of care, in which low-risk pregnant women are supported by a midwife during vaginal childbirth. There should be development of the educational status, in the Bachelor’s of midwifery degree students and in graduates, about RMC to let them gain experience and communication skills in the educational hospitals.

## Data Availability

The data supporting this research are available from the authors on reasonable request.
